# Recurrence of Retinopathy of Prematurity Following Anti-vascular Endothelial Growth Factor (Anti-VEGF) Therapy: A Systematic Review and Meta-Analysis

**DOI:** 10.7759/cureus.73286

**Published:** 2024-11-08

**Authors:** Mohammed Dablouk, Amit Chhabra, Ahmed T Masoud

**Affiliations:** 1 Internal Medicine, Mersey and West Lancashire Teaching Hospitals NHS Trust, Merseyside, GBR; 2 Ophthalmology, Lancashire Teaching Hospitals NHS Foundation Trust, Preston, GBR; 3 Epidemiology and Public Health, Fayoum University, Faiyum, EGY

**Keywords:** anti-vegf therapy, laser photocoagulation, recurrence, retinopathy of prematurity, retreatment

## Abstract

Retinopathy of prematurity (ROP) is a neovascular disorder which affects premature infants and can lead to childhood blindness. Treatment includes laser photocoagulation and intravitreal anti-vascular endothelial growth factor (anti-VEGF) injections, both effective options. However, laser photocoagulation can lead to refractive errors, while anti-VEGF therapy can result in disease recurrence. Our systematic review aimed to evaluate the recurrence rates of ROP following treatment with anti-VEGF agents compared to other anti-VEGF agents and laser photocoagulation. We also aimed to measure the retreatment intervals and assess successful retreatment rates to identify the most effective treatment modality.

Our review adhered to the Preferred Reporting Items for Systematic Reviews and Meta-Analyses (PRISMA) guidelines and included both randomized controlled trials (RCTs) and non-randomized comparative studies (NCS). A thorough search of MEDLINE, EMBASE, Web of Science, and Cochrane Central Register of Controlled Trials (CENTRAL) databases was performed. Studies were assessed using the Cochrane Risk of Bias (RoB2) tool and the Newcastle-Ottawa Scale (NOS). Random-effects and fixed-effects models were used to measure pooled estimates. The outcomes were reported as risk ratios (RR) and standardized mean differences (SMD).

A total of 21 studies (six RCTs and 15 NCS) with 6,152 eyes were included. Overall, anti-VEGF agents showed a higher risk of recurrence compared to laser photocoagulation (RR=2.14, 95% CI: 1.06-4.33, p=0.03). Conbercept demonstrated a significantly lower risk of recurrence than laser photocoagulation and ranibizumab (RR=0.47, 95% CI: 0.39-0.58, p<0.00001), while aflibercept showed a higher recurrence risk compared to bevacizumab (RR=12.61, 95% CI: 6.43-24.73, p<0.00001). Bevacizumab was associated with a longer retreatment interval than laser photocoagulation (SMD=0.89, 95% CI: 0.61-1.17, p<0.00001). No significant differences in retreatment success rates between bevacizumab and laser photocoagulation were observed.

Anti-VEGF therapy is effective in the treatment of ROP; however, it is associated with a higher risk of recurrence compared to laser photocoagulation. Conbercept shows greater efficacy in reducing recurrence risk and prolonging the retreatment interval compared to ranibizumab. Aflibercept demonstrated inconsistent outcomes. No significant difference in recurrence rates was observed between bevacizumab and ranibizumab. Recurrences occurred significantly later when anti-VEGF therapy was used compared to laser photocoagulation. Successful retreatment rates between bevacizumab and laser photocoagulation were similar.

## Introduction and background

Retinopathy of prematurity (ROP) is a vaso-proliferative disorder primarily affecting preterm, low birth weight infants. In ROP, the abnormal development of retinal blood vessels results in the neovascularization of the inner retina, making it one of the most preventable causes of childhood blindness globally [1]. Infants born at or before 30 weeks gestation or with a birth weight of ≤1,500 g are at the highest risk [2]. The incidence and severity of ROP are inversely related to both gestational age and birth weight [3]. If left untreated, ROP can lead to complications such as refractive errors, anatomical abnormalities, and retinal detachment, which may result in permanent vision loss.

Retinal vascularization starts around 12 weeks of gestation [4]. While most infants follow normal retinal development postnatally, preterm infants may develop ROP, which has a biphasic disease course: an initial vaso-obliterative phase followed by a vaso-proliferative phase [5].

Phase I

From birth to 30-32 weeks postmenstrual age (PMA), retinal vessel growth halts, causing immature vessel obliteration due to vascular endothelial growth factor (VEGF) suppression from hyperoxia (oxygen supplementation) and low retinal metabolic demand [6,7]. Low insulin-like growth factor 1 (IGF-1), secondary to poor nutrition or sepsis, further impairs VEGF activation [8]. As metabolic demand increases, hypoxia stimulates VEGF and IGF-1 production, initiating phase II [9].

Phase II

This phase involves hypoxia-driven retinal neovascularization. Before 32 weeks, low metabolic demand limits hypoxia, but as it rises, VEGF upregulation leads to abnormal vessel growth [10,11]. A ridge forms between vascularized and avascular areas, with some cases returning to normal vessel development and others developing fragile, poorly perfused capillaries, which worsen hypoxia and abnormal growth [12], leading to ROP.

The International Committee for the Classification of Retinopathy of Prematurity (ICROP) has developed a diagnostic system to classify ROP based on location and severity. This classification provides a standardized framework for identifying and managing the disease [13,14]. Regarding location, ROP is classified into three concentric retinal zones centered on the optic disc and extending outward to the ora serrata. As ROP progresses and becomes more severe, specific vascular features develop and are categorized into different stages [15].

Initially, cryotherapy was used for ROP treatment, but laser photocoagulation replaced it as the dominant therapy over the past three decades, showing benefits for type 1 ROP in specific zones, as highlighted by the Early Treatment for Retinopathy of Prematurity (ETROP) study [16,17]. However, laser therapy carries risks such as myopia, retinal damage, and sometimes retinal detachment [18,19]. With advancements in understanding ROP pathology and the efficacy of VEGF inhibitors like bevacizumab in other eye diseases, such as in diabetic retinopathy [20] and age-related macular degeneration [21], anti-VEGF drugs became an option for ROP treatment. The Bevacizumab Eliminates the Angiogenic Threat of Retinopathy of Prematurity (BEAT-ROP) study found bevacizumab to be more effective than laser therapy for zone I stage 3 disease [22], but this benefit was not seen in zone II disease. Further studies, such as the Ranibizumab Versus Laser Therapy for the Treatment of Very Low Birth Weight Infants with Retinopathy of Prematurity (RAINBOW) trial, suggested ranibizumab might provide better outcomes with fewer side effects compared to laser treatment [23]. A recent meta-analysis indicated that aflibercept is similarly effective to other anti-VEGF agents [24]. Newer treatments, including conbercept and pegaptanib, show promise, particularly in combination with laser therapy for advanced ROP [25-27].

Anti-VEGF therapy has transformed ROP management by inducing rapid disease regression. However, recurrence remains a significant concern, frequently observed between 44 and 55 weeks PMA [28,29]. Recurrence may present as new demarcation lines or more severe forms like stage 3 ROP, potentially leading to complications such as fibrosis and tractional detachment. Although anti-VEGF therapy is increasingly used, questions remain about its long-term effects, recurrence risks, and optimal retreatment strategies. Recurrences generally occur between 37 and 60 weeks PMA, influenced by factors like the anti-VEGF agent used, dosage, patient characteristics, or reinjections [30-32]. Not all recurrences require treatment; decisions are often based on clinical judgment [33,34]. Additionally, recurrences may occur significantly later, particularly if reinjections are used [35].

The primary aim of our review was to evaluate the relationship between ROP recurrence and anti-VEGF treatment. This included comparing the recurrence rates after anti-VEGF therapy with those following laser photocoagulation and other anti-VEGF agents. Furthermore, we examined the time interval between initial treatment and retreatment to identify any significant differences. Lastly, we assessed the success rates of various retreatment options to determine the most effective approach for managing ROP recurrence.

## Review

Methodology

We conducted a comprehensive systematic review to assess ROP recurrence following intravitreal anti-VEGF therapy. Our review followed the Preferred Reporting Items for Systematic Reviews and Meta-Analyses (PRISMA) 2020 guidelines. A meta-analysis was performed to synthesize comparable studies and draw robust conclusions about the review's outcomes, with professional statistical advice being sought to ensure accuracy. The population, intervention, comparator, outcome, and study design (PICOS) framework was employed to structure the research question and identify relevant data and key characteristics. Our review focused on infants who were born prematurely and diagnosed with ROP (Table 1). Management included a single intravitreal anti-VEGF agent. The agents included were bevacizumab, ranibizumab, aflibercept, and conbercept. Comparisons were made between anti-VEGF agents and laser photocoagulation and between different anti-VEGF agents. However, studies using the same agent but with different dosages, frequencies, or timing were excluded (Table 2). Furthermore, studies involving combination treatment were excluded. The primary outcome of interest was disease recurrence following anti-VEGF therapy. Adverse effects, refractive outcomes, and mortality were not relevant to this review. Only studies that reported recurrence data were included in the final analysis. While randomized controlled trials (RCTs) offer the highest level of evidence and were prioritized, non-randomized comparative studies (NCS) were also included due to the limited data on ROP recurrence, acknowledging their higher potential for bias.

**Table 1:** Inclusion criteria ROP: retinopathy of prematurity; anti-VEGF: anti-vascular endothelial growth factor; NCS: non-randomized comparative studies; RCT: randomized controlled trials

Criteria	Inclusion details
Population	Infants less than 34 weeks gestation and infants weighing less than 2000 g, diagnosed with ROP
Intervention	Intravitreal anti-VEGF monotherapy
Comparator	Laser photocoagulation therapy or other intravitreal anti-VEGF agents
Outcome	Rate of disease recurrence (studies must report this outcome), retreatment/recurrence interval, retreatment outcome
Study design	RCT and NCS

**Table 2:** Exclusion criteria ROP: retinopathy of prematurity; anti-VEGF: anti-vascular endothelial growth factor

Criteria	Exclusion details
Population	Infants more than 34 weeks gestation, infants weighing more than 2000 g, infants who received surgical treatment for ROP, infants with vitreoretinal conditions other than ROP, animal studies
Intervention	Treatment modalities other than intravitreal anti-VEGF agents or laser photocoagulation
Comparator	Treatment modalities other than intravitreal anti-VEGF agents or laser photocoagulation or the same anti-VEGF agent
Outcome	Local adverse effects, systemic adverse effects, refractive outcomes (long and short term), neurodevelopmental effects, mortality
Study design	Non-comparative studies, single-arm studies, systematic reviews, non-English studies, unavailability of full text, article reviews

Search Strategy

The search strategy utilized four major bibliographic databases: EMBASE (1980-week 12/22) and MEDLINE (1980-week 12/22) (see Appendices), both accessed via the Ovid interface, as well as the Cochrane Central Register of Controlled Trials (CENTRAL) and Web of Science (all years). Citation chaining of included studies and related systematic reviews was conducted to identify additional relevant studies. Initially, EndNote 20 (Clarivate, London, United Kingdom) was used as the primary reference management tool, but Covidence (Veritas Health Innovation, Melbourne, Australia) later replaced it for its more user-friendly interface. The same search was replicated with Covidence, ensuring all dates matched the initial search timeline. A second search was performed before the final analysis (week 23/22) to include any newly published studies. Covidence was employed to screen and review studies [36]. Two reviewers independently screened the search results by titles and abstracts for relevance. The Covidence interface allowed reviewers to select "Yes", "No", or "Maybe" for each study. Results could only be viewed after both reviewers completed the screening, with any disagreements flagged for discussion in a separate section. A study was included if both reviewers agreed with "Yes" or after resolving disagreements through discussion. Full-text reviews were conducted in the following cases: studies marked as "Maybe", unresolved disagreements after the title and abstract screening, and studies deemed potentially eligible.

Data Collection

Two reviewers independently extracted data from the included studies. Disagreements were highlighted and discussed after completing data extraction. The primary outcomes of interest were recurrence rate, recurrence interval, and successful retreatment. Relevant data items included sample size (number of eyes), PMA at initial treatment, recurrence time after initial treatment (in weeks), PMA at recurrence/retreatment, retreatment, and successful retreatment rates. Missing data were addressed by contacting the respective study authors. If no response was received within two weeks, a decision was made based on the availability of recurrence data. Studies with recurrence data were included and analyzed, but those without were excluded.

Statistical Synthesis

For the meta-analysis, we utilized RevMan (Review Manager, Version 5.4, The Cochrane Collaboration, London, England, United Kingdom). A random-effects model was used to account for the clinical and methodological differences among the studies. Statistical significance was determined at a p-value less than 0.05, and 95% confidence intervals (CI) were included throughout the analysis. To assess variability between and within studies, heterogeneity was measured using both the I² statistic and the p-value from the chi-squared test. Outcomes like recurrence rates and retreatment success were reported as risk ratios (RR), while retreatment intervals were expressed as standardized mean differences (SMD). This approach allowed for the pooling of results across studies. Heterogeneity was categorized based on I² values, where values more than 75% suggested considerable heterogeneity, 50-75% substantial heterogeneity, 25-50% moderate heterogeneity, and below 25% minimal heterogeneity. In the early phases, we used OpenMeta Analyst [37] minimally for certain calculations. Additional analyses were performed separately when some studies did not provide important metrics such as standard deviation [38].

Study Characteristics

Our systematic review identified six eligible RCTs [22,25,33,34,39,40], with a combined sample size of 470 infants and 940 eyes, focusing on the treatment outcomes of ROP. These studies were conducted between 2016 and 2022. Three of the five RCTs that included photocoagulation as a treatment arm explicitly stated that their studies were non-masked, and due to the visible nature of laser burn marks, all five studies were effectively non-masked. The mean birth weight of infants ranged from 615.2 g to 1273 g, with gestational ages between 24.2 weeks and 28.96 weeks. Follow-up durations varied, with a minimum period of six months, which is critical for assessing long-term outcomes such as recurrence and complications. The primary outcomes measured across the studies included recurrence, regression, and complication rates. Additionally, 15 NCS [26,41-54] were included, with a total of 2709 infants and 5176 eyes. These studies were conducted between 2014 and 2022. Most were retrospective, using chart reviews to collect data on patient demographics, treatment protocols, and clinical outcomes. Four of the 15 studies involved three intervention arms, while the remaining 11 included two intervention arms. Mean birth weights ranged from 629.9 g to 2263.4 g, and gestational ages ranged between 24 weeks and 32.1 weeks. Follow-up periods varied but were at least six months, except for Gunay et al. [47], which had a shorter follow-up duration. Outcomes measured included recurrence, regression, complications, and both refractive and anatomical outcomes, offering a comprehensive assessment of the efficacy and safety of the interventions studied.

Quality Assessment

**Figure 1:**
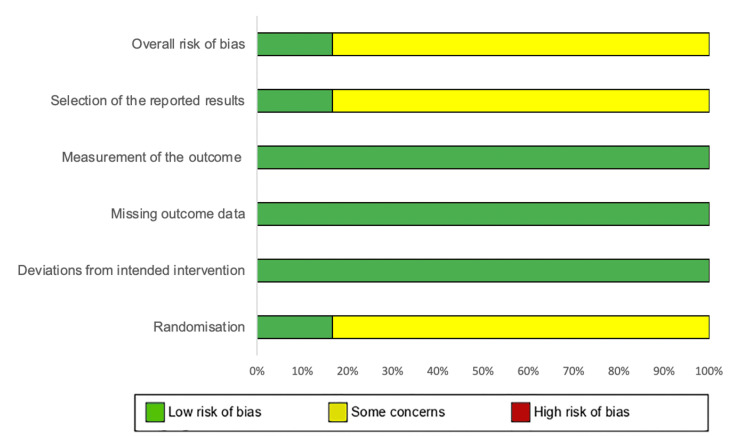
RoB graph for the evaluation of RCTs using the RoB2 tool RCT: randomized controlled trial; RoB: risk of bias

**Figure 2:**
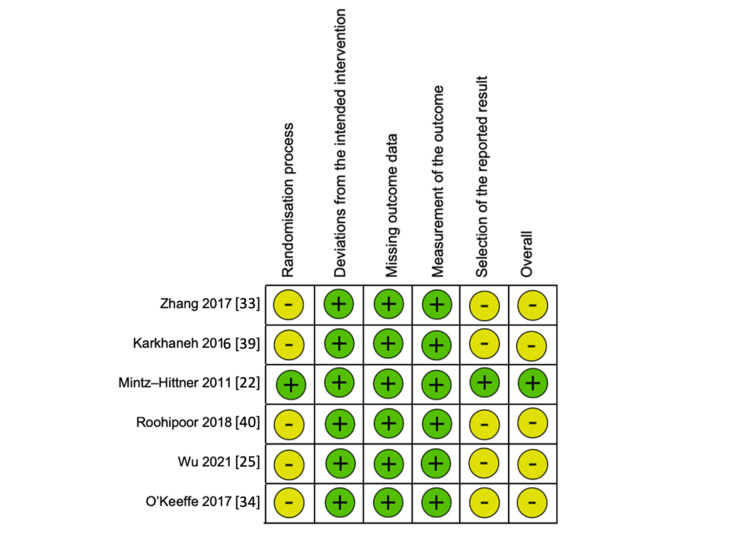
RoB summary for the evaluation of RCTs using the RoB2 tool RCT: randomized controlled trial; RoB: risk of bias

The risk of bias was assessed using two recognized tools: the Newcastle-Ottawa Scale (NOS) [55] for non-randomized studies and version 2 of the RoB2 tool [56] for RCTs. The NOS examines three critical categories: selection (four items), comparability (one item), and exposure (three items). Each study can receive one star per item in the selection and exposure categories, while a maximum of two stars can be awarded for comparability (Table 3). Studies were classified, based on the total score, as low quality (0-3 stars), moderate quality (4-6 stars), or high quality (7-9 stars). The RoB2 tool assesses potential sources of bias across five domains in RCTs (Figure 1 and Figure 2). This includes bias arising from the randomization process, bias due to deviations from intended interventions, bias due to missing outcome data, bias in the measurement of outcomes, and bias in the selection of reported results. Fifteen non-randomized studies were assessed using the NOS. Eleven studies were rated as high quality (seven studies scored 7 stars, and four scored 8 stars), while four studies were rated as moderate quality (one scored 5 stars, and three scored 6 stars). Six RCTs were evaluated using the RoB2 tool. One study, Mintz-Hittner et al. [22], was rated as low risk of bias. The remaining five studies were assessed as having "some concerns," mainly because of potential biases in the randomization process and the selection of reported results, which may affect the reliability of their findings.

**Table 3:** Quality assessment of NCS using the NOS NCS: non-randomized comparative studies; NOS: Newcastle-Ottawa Scale

Study	Selection				Comparability	Exposure			Overall score
	Is the case definition adequate?	Representativeness of the cases	Selection of controls	Definition of controls	Comparability of cases and controls based on design or analysis	Ascertainment of exposure	Same method of ascertainment for cases and controls	Non-response rate	
Linghu et al. 2022 [41]	★	★	-	★	★★	★	★	★	8
Sukgen and Koçluk 2019 [42]	★	★	-	-	★★	★	★	★	7
Jin et al. 2018 [26]	★	-	-	★	★	★	★	★	6
Riazi-Esfahani et al. 2021 [43]	★	-	-	★	★★	★	★	★	7
Gunay et al. 2015 [44]	★	★	-	-	★★	★	★	★	7
Cheng et al. 2020 [45]	★	-	-	★	★★	★	★	★	7
Süren et al. 2022 [46]	★	★	-	★	★★	★	★	★	8
Gunay et al. 2017 [47]	★	★	-	★	★★	★	★	★	8
Moran et al. 2014 [48]	★	-	-	★	★	★	★	-	5
Isaac et al. 2015 [49]	★	-	-	★	★	★	★	★	6
Hwang et al. 2015 [50]	★	★	-	★	★★	★	★	-	7
Iwahashi et al. 2021 [51]	★	-	-	★	★	★	★	★	6
Kabataş et al. 2017 [52]	★	★	-	-	★★	★	★	★	7
Eftekhari Milani et al. 2022 [53]	★	★	-	★	★★	★	★	★	8
Kang et al. 2018 [54]	★	★	-	★	★	★	★	★	7

Results

**Figure 3:**
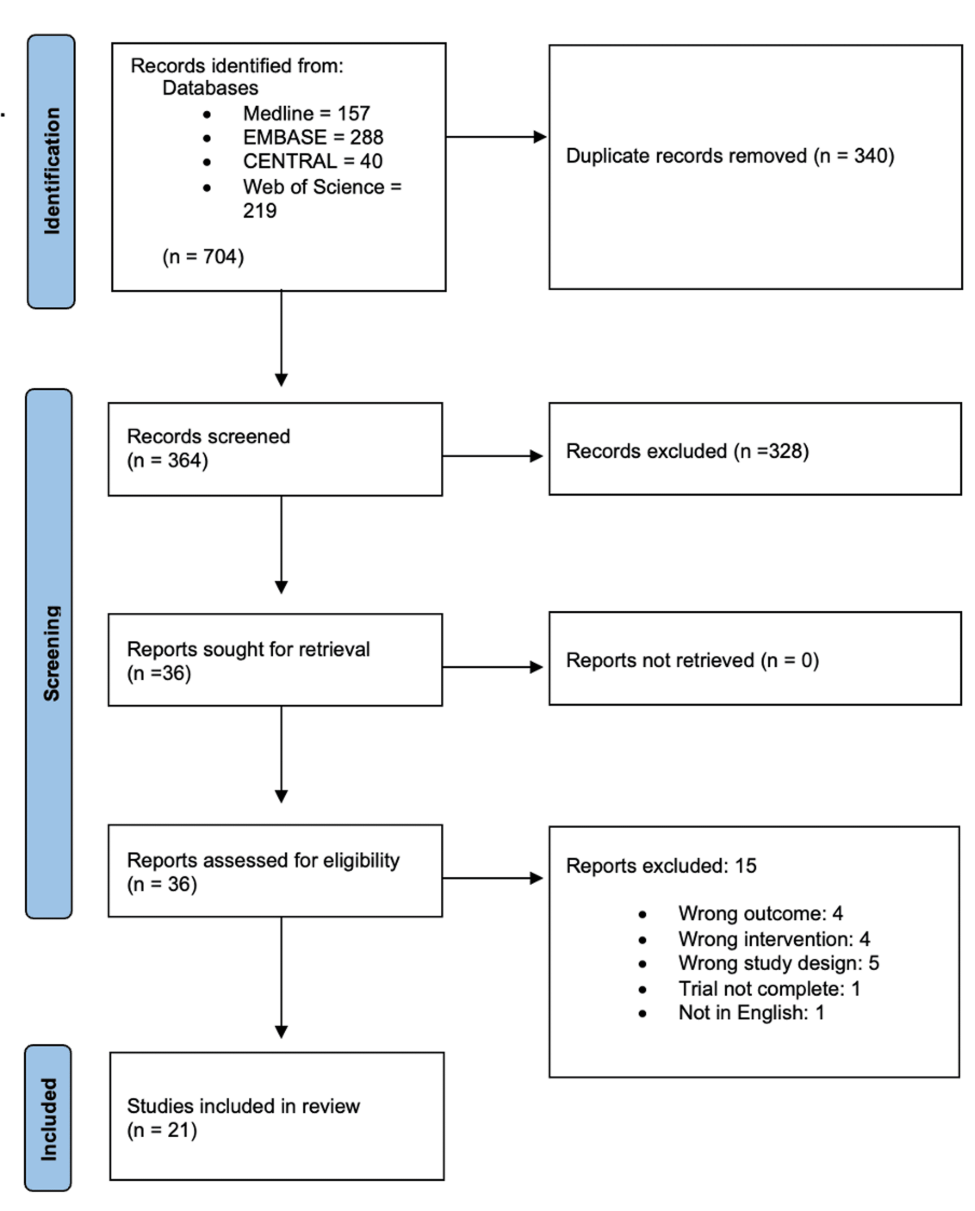
PRISMA flow diagram PRISMA: Preferred Reporting Items for Systematic Reviews and Meta-Analyses

The PRISMA flow diagram visually summarizes the screening process, showing the progression from 704 initially identified records to the 21 studies included in the final review. The diagram outlines each step, including the removal of duplicates, exclusions based on screening criteria, and the reasons for excluding certain studies (Figure 3).

The study characteristics are summarized in the two tables below. The first table includes RCTs (Table 4) and the following table includes the NCS (Table 5). The tables help illustrate the diversity of study designs and the range of populations included in the review.

**Table 4:** Characteristics of the included randomized controlled trials Bev: bevacizumab; Rani: ranibizumab; Conb: conbercept; PMA: postmenstrual age; ROP: retinopathy of prematurity

Author and year	Study design	Intervention	Number of participants (eyes)	Mean birth weight (g)	Gestational age (weeks)	Number of eyes (anti-VEGF)	Number of eyes (lasers)	Follow-up	Primary outcome
Karkhaneh et al. 2016 [39]	Randomized controlled non-masked prospective study	Bevacizumab vs laser photocoagulation	79 (158)	Bev: 1133±344	Bev: 28.37±1.96	86	72	Until 90 weeks PMA	ROP persistence/recurrence
				Laser: 1202±321	Laser: 28.50±1.99				
Zhang et al. 2017 [33]	Randomized controlled prospective single-center study	Ranibizumab vs laser photocoagulation	50 (100)	Rani: 1220±320	Rani: 28.96±1.59	50	50	At least 6 months	Disease regression
				Laser: 1060±240	Laser: 28.27±1.84				ROP recurrence
Mintz-Hittner et al. 2011 [22]	Randomized controlled non-masked stratified multicenter study	Bevacizumab vs laser photocoagulation	150 (300)	Bev (ZI): 615.2±139.5	Bev (ZI): 24.2±1.3	(ZI) 66	(ZI) 68	Until 54 weeks PMA	Recurrence of ROP requiring retreatment before 54 weeks PMA
				Bev (ZII): 689.2±111.3	Bev (ZII): 24.5±1.2				
				Laser (ZI): 657.9±159.5	Laser (ZI): 24.3±1.6	(ZII) 84	(ZII) 82		
				Laser (ZII): 680.7±156.8	Laser (ZII): 24.5±1.4				
Roohipoor et al. 2019 [40]	Randomized controlled non-masked prospective study	Bevacizumab vs laser photocoagulation	116 (232)	Bev: 1232±318	Bev: 28.75±1.86	154	78	Until 90 weeks PMA	Rate of ROP regression
				Laser: 1273±273	Laser: 28.32±2.11				
Wu et al. 2022 [25]	Randomized controlled prospective multicenter study	Conbercept vs ranibizumab	60 (120)	Conb: 1160±480	Conb: 28.27±2.77	60	60	6 months	Disease regression
				Rani: 1089±400	Rani: 27.50±2.70				ROP recurrence
O'Keeffe et al. 2016 [34]	Randomized controlled prospective study	Bevacizumab vs laser photocoagulation	15 (30)	Bev: 795.4±181.1	Bev: 25.7±1.28	15	15	5 years	Disease regression
									Visual outcomes

**Table 5:** Characteristics of the included non-randomized studies Anti-VEGF: anti-vascular endothelial growth factor; Rani: ranibizumab; Afli: aflibercept; Conb: conbercept; Bev: bevacizumab; ROP: retinopathy of prematurity

Author and year	Intervention	Number of participants (eyes)	Mean birth weight (g)	Gestational age (weeks)	Number of eyes (anti-VEGF/laser)	Follow-up	Outcome
Linghu et al. 2022 [41]	Bevacizumab vs laser photocoagulation vs ranibizumab vs conbercept	862 (1627)	Anti-VEGF (ZI): 1334.7±374.9	Anti-VEGF (ZI): Bev: 29.5±2.1	Bev: 25	6 months	Rate of initial regression
			Anti-VEGF (ZII): 1252.5±368.4	Anti-VEGF (ZII): 28.7±2.0	Rani: 916		
			Laser (ZI): 1282.2±258.9	Laser (ZI): 29.2±2.1	Con: 283		Reactivation requiring retreatment
			Laser (ZII) 1410.9±374.6	Laser (ZII): 30.2±2.3	Laser: 403		Refractive status
Sukgen and Koçluk 2019 [42]	Ranibizumab vs aflibercept	63 (126)	Rani: 1126.7±386.7	Rani: 28.35±2.05	Rani: 54	1 year	Regression of disease
			Afli: 1157.6±298.2	Afli: 28.3±2.05	Afli: 72		
							Disease recurrence
							Ocular complications
Jin et al. 2018 [26]	Ranibizumab vs conbercept	24 (48)	Conb: 1369±161.9	Conb: 29.49±1.37	Conb: 28	9.8 weeks	Regression of plus disease
			Rani: 1171.4±279.9	Rani: 28.35±1.62	Rani: 20		Recurrence of plus disease
Riazi-Esfahani et al. 2021 [43]	Bevacizumab vs aflibercept	453 (889)	Bev: 1119±311	Bev: 28.2±2	Bev: 865	Bev: 289±257 days	Rate and time of complete regression
			Afli: 1205±383	Afli: 28.7±2.3	Afli: 24	Afli: 143±25 days	Recurrence rates
Gunay et al. 2015 [44]	Bevacizumab vs laser photocoagulation	40 (78)	Bev: 901.4±304.6	Bev: 26.4±1.82	Bev: 48	2 years	Clinical outcomes
			Laser: 941±282.48	Laser: 27.3±1.82	Laser: 30		Refractive outcomes
Cheng et al. 2020 [45]	Conbercept vs ranibizumab	625 (1199)	Conb (ZI): 1189.4±320.7	Conb (ZI): 28±2.3	Conb: 916	1 year	Regression
			Conb (ZII): 2198.5±290.6	Conb (ZII): 32±1.7			Progression
			Rani (ZI): 1193.4±306.1	Rani (ZI): 27.3±1.8	Rani: 283		
			Rani (ZII): 2263.4±289.2	Rani (ZII): 28±2.3			Recurrence
Süren et al. 2022 [46]	Bevacizumab vs ranibizumab vs aflibercept	111 (187)	Bev: 1060±539	Bev: 27±2.82	Bev: 54	3 years	Recurrence
			Rani: 1066±313	Rani: 28±2.5	Rani: 77		Refractive outcomes
			Afli: 984±539	Afli: 27±2.11	Afli: 56		
Gunay et al. 2017 [47]	Bevacizumab vs ranibizumab vs laser photocoagulation	134 (268)	Bev: 1005.29±411.19	Bev: 27.31±2.18	Bev: 110	Bev: 19.40±6.43 months	Total inactivation of ROP
			Rani: 1195.9±466.98	Rani: 27.95±2.90	Rani: 44	Rani: 18.96±4.79 months	
			Laser: 1119.47±336.96	Laser: 28.23±2.5	Laser: 114	Laser: 20.68±6.89 months	Refractive outcomes at 1.5 years
Moran et al. 2014 [48]	Bevacizumab vs laser photocoagulation	14 (28)	Bev: 722±131	Bev: 25.2±1.4	Bev: 14	2 years	Recurrence of ROP
			Laser: 674±175	Laser: 25±1.1	Laser: 14		Visual outcomes
Isaac et al. 2015 [49]	Bevacizumab vs laser photocoagulation	25 (45)	Bev: 722±131	Bev: 25.2±1.4	Bev: 23	1 year	Structural outcomes
			Laser: 674±175	Laser: 25±1.1	Laser: 22		Visual function
Hwang et al. 2015 [50]	Bevacizumab vs laser photocoagulation	28 (54)	Bev (ZI): 667.6±117.4	Bev (ZI): 24.3±1	Bev: 22	Bev: 21.7±8.8 months	Recurrence
			Bev (ZII): 669.3±181.2	Bev (ZII): 24±1			
			Laser (ZI): 697.7±89.6	Laser (ZI): 24.4±0	Laser: 32	Laser: 34.5±20.4 months	Complication rate
			Laser (ZII): 702.2±127	Laser (ZII): 24.9±1.3			Refractive error
Iwahashi et al. 2021 [51]	Bevacizumab vs ranibizumab	43 (80)	Bev: 629.9±216.2	Bev: 24.7±1.5	Bev: 37	Bev: 39±15 months	Clinical outcomes of anti-VEGF therapy
			Rani: 714.1±291.7	Rani: 25±2.2	Rani: 43	Rani: 10.6±6.6 months	
Kabataş et al. 2017 [52]	Bevacizumab vs ranibizumab vs laser photocoagulation	54 (108)	Bev: 841±235	Bev: 26.1±2.27	Bev: 24	18 months	Recurrence rates
			Rani: 840±177	Rani: 26±1.26	Rani: 12		Refractive status
			Laser: 1112±362	Laser: 27.7±2.7	Laser: 72		Complications
Eftekhari Milani et al. 2022 [53]	Bevacizumab vs aflibercept	150 (286)	Bev: 1060.38±280.8	Bev: 28.52±2.1	Bev: 210	3 years	Treatment outcomes
			Afli: 1031.05±211.5	Afli: 28.89±2.0	Afli: 56		
Kang et al. 2018 [54]	Bevacizumab vs ranibizumab	83 (153)	Bev: 941.8±296.1	Bev: 26.9±1.9	Bev: 101	25.1±1.5 months	Recurrence rate
			Rani: 1257.7±514.5	Rani: 28.1±3.2	Rani: 52		Refractive error

Recurrence Rate Between Anti-VEGF Agents and Laser Photocoagulation

**Figure 4:**
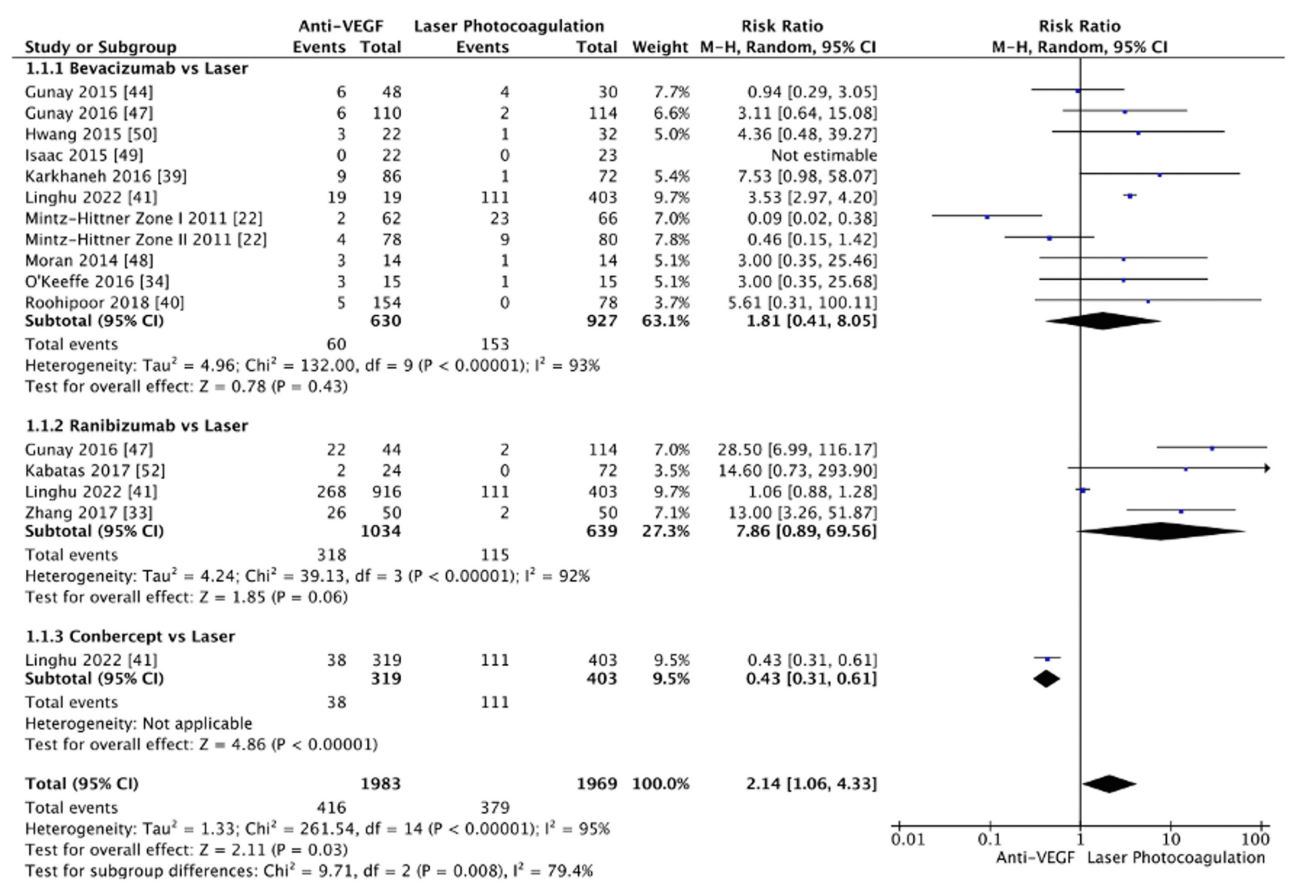
Recurrence rate, subgroup bevacizumab, ranibizumab, and conbercept vs laser

**Figure 5:**
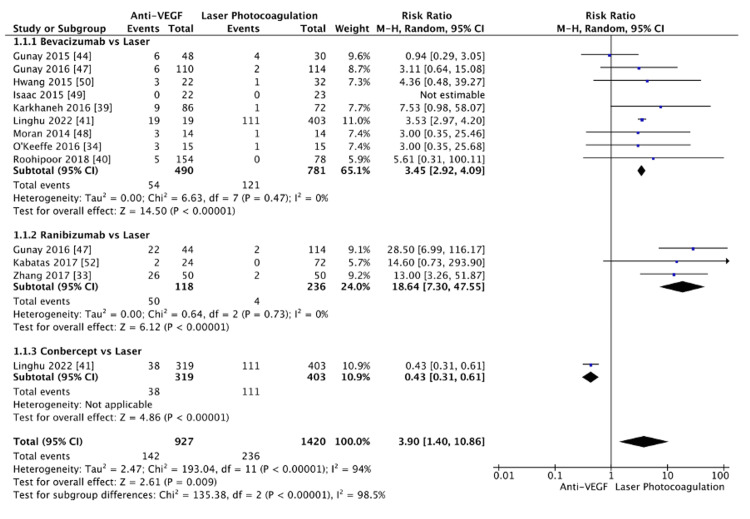
Recurrence rate, post-sensitivity analysis

**Figure 6:**
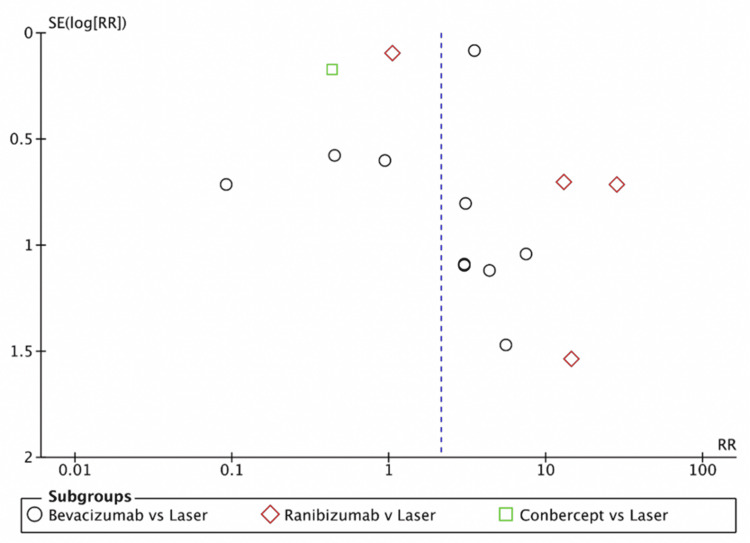
Funnel plot of recurrence rate

A subgroup analysis compared the recurrence rates between intravitreal anti-VEGF agents (bevacizumab, ranibizumab, conbercept) and laser photocoagulation (Figure 4). In the bevacizumab versus laser subgroup, no significant difference was observed in recurrence rate (RR=1.81, 95% CI: (0.41, 8.05), p=0.43), with high heterogeneity (I²=93%) noted. Similarly, the ranibizumab versus laser comparison also showed no significant difference (RR=7.86, 95% CI: (0.89, 69.56), p=0.06, I²=92%). However, in the conbercept versus laser subgroup, a significantly lower risk of recurrence was found with conbercept compared to laser photocoagulation (RR=0.43, 95% CI: (0.31, 0.61), p<0.00001). Notably, this finding is based on a single large study (n=722). When pooling data from all anti-VEGF agents, the analysis indicated a statistically significant increase in recurrence risk with anti-VEGF therapy compared to laser photocoagulation (RR=2.14, 95% CI: (1.06, 4.33), p=0.03). However, substantial heterogeneity was observed (I²=95%). Sensitivity analyses were conducted to identify sources of this heterogeneity. In the bevacizumab subgroup, removing the Mintz-Hittner et al.'s study [22] resolved the heterogeneity (I²=0%) and revealed a significant increase in recurrence risk for bevacizumab versus laser photocoagulation (RR=3.45, 95% CI: (2.92, 4.09), p<0.00001). Similarly, excluding Linghu et al.'s study [41] from the ranibizumab subgroup eliminated heterogeneity (I²=0%) and revealed a significantly higher recurrence rate with ranibizumab (RR=18.64, 95% CI: (7.30, 47.55), p<0.00001). After the removal of Mintz-Hittner et al. [22] and Linghu et al. [41] from the pooled analysis, the overall effect size remained significant (RR=3.90, 95% CI: (1.40, 10.86), p<0.00001), further confirming the increased risk of recurrence with anti-VEGF agents compared to laser photocoagulation (Figure 5). While the sensitivity analysis successfully resolved heterogeneity within the subgroups, the overall heterogeneity across subgroups remained high (I²=98.5%), likely reflecting the real clinical differences between the anti-VEGF agents regarding recurrence rates. A funnel plot was generated to assess potential publication bias among the included studies (Figure 6). The plot exhibited some asymmetry, with a rightward skew suggesting higher recurrence rates with anti-VEGF agents than laser photocoagulation. While this could indicate a potential for bias, it is more likely reflective of the inherent differences between the included studies.

Comparison Between Intravitreal Anti-VEGF Agents

A random-effects model was used to compare the recurrence rates of ROP between different intravitreal anti-VEGF agents. These comparisons included conbercept versus ranibizumab, aflibercept versus ranibizumab, aflibercept versus bevacizumab, and bevacizumab versus ranibizumab.

**Figure 7:**
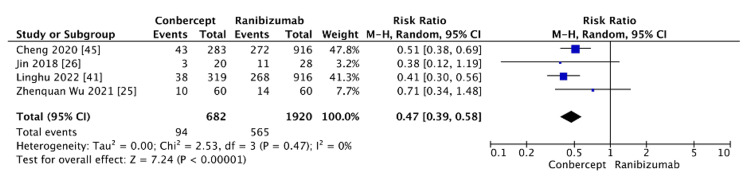
Recurrence rate between conbercept and ranibizumab

Conbercept vs. ranibizumab: The comparison between conbercept and ranibizumab showed a significant reduction in the risk of recurrence with conbercept (RR=0.47, 95% CI: 0.39-0.58, p<0.00001) (Figure 7). There was no evidence of heterogeneity (I²=0%).

**Figure 8:**
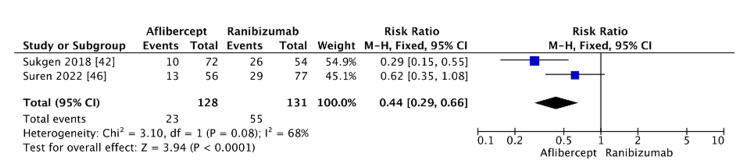
Recurrence rate between aflibercept and ranibizumab

Aflibercept vs. ranibizumab: A statistically significant difference was observed between aflibercept and ranibizumab, with a lower risk of recurrence associated with aflibercept (RR=0.44, 95% CI: 0.29-0.66, p<0.0001) (Figure 8). Moderate heterogeneity was detected (I²=68%). This level of heterogeneity warrants caution in interpretation, particularly given the small number of included studies. The I² statistic can be sensitive to small sample sizes, and minor differences in study weight may contribute disproportionately to the I² value, leading to an overestimation of heterogeneity.

**Figure 9:**
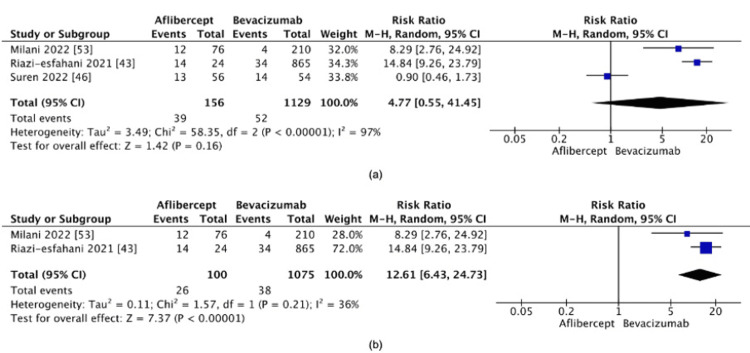
(a) Recurrence rate between aflibercept and bevacizumab. (b) Post-sensitivity analysis of the recurrence rate between aflibercept and bevacizumab

Aflibercept vs. bevacizumab: The analysis comparing aflibercept and bevacizumab showed no significant difference in recurrence rates (RR=4.77, 95% CI: 0.55-41.45, p=0.16) (Figure 9a), suggesting that neither agent exhibited a distinct advantage in this population. However, high heterogeneity (I²=97%) raised concerns about the estimate's reliability. A sensitivity analysis was performed by excluding the study by Süren et al. [46], which was identified as the primary contributor to the heterogeneity. After exclusion, heterogeneity decreased significantly (I²=36%), and the pooled effect size indicated a higher risk of recurrence with aflibercept (RR=12.61, 95% CI: 6.43-24.73, p<0.00001) (Figure 9b).

**Figure 10:**
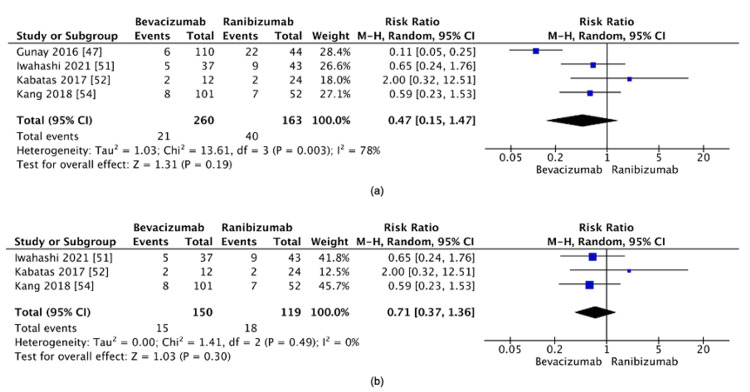
(a) Recurrence rate between bevacizumab and ranibizumab. (b) Post-sensitivity analysis of the recurrence rate between bevacizumab and ranibizumab

Bevacizumab vs. ranibizumab: The comparison between bevacizumab and ranibizumab revealed no statistically significant difference in recurrence rates (RR=0.47, 95% CI: 0.15-1.47, p=0.19) (Figure 10a). Considerable heterogeneity was observed (I²=78%). A sensitivity analysis excluding Gunay et al. [27] resolved the heterogeneity (I²=0%), but the result remained non-significant (RR=0.71, 95% CI: 0.37-1.36, p=0.30) (Figure 10b). These results suggest that the lack of a significant difference between bevacizumab and ranibizumab is robust and not merely due to variability across studies.

Time Comparisons Between Initial Treatment and Retreatment

A random-effects model was applied to assess the time from initial treatment to retreatment, using the SMD as the effect measure. Although the hazard ratio would have been the preferred measure for retreatment intervals, it was not reported in the included studies. Furthermore, the lack of individual patient data prevented us from calculating the hazard ratios. Thus, the SMD was chosen as an appropriate alternative to compare retreatment intervals across the included studies.

**Figure 11:**
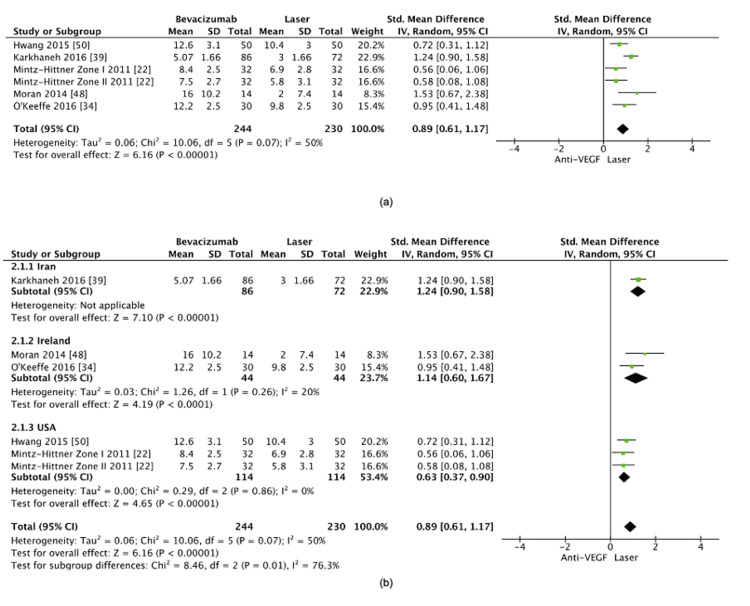
(a) Retreatment interval between bevacizumab and laser photocoagulation. (b) Post-sensitivity analysis of the retreatment interval between bevacizumab and laser photocoagulation

Bevacizumab vs. laser photocoagulation: The comparison between bevacizumab and laser photocoagulation revealed a statistically significant difference in the time to retreatment, with bevacizumab being associated with a longer retreatment interval (SMD=0.89, 95% CI: 0.61-1.17, p<0.00001) (Figure 11a). Moderate heterogeneity was observed (I²=50%). A sensitivity analysis was performed to explore potential sources of heterogeneity, focusing on factors such as study design, sample size, and geographic location. Karkhaneh et al. [39] was identified as the primary source of heterogeneity. When stratifying studies based on geographic location, namely, Ireland, the United States, and Iran, the sensitivity analysis yielded a more homogeneous dataset, and the pooled effect remained statistically significant (SMD=0.76, 95% CI: 0.51-1.02, p<0.00001) (Figure 11b). All subgroups demonstrated a longer retreatment interval with bevacizumab, though the magnitude of this difference varied.

**Figure 12:**
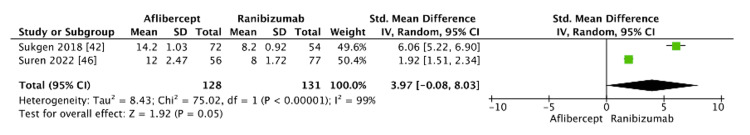
Retreatment interval between aflibercept and ranibizumab

Aflibercept vs. ranibizumab: No statistically significant difference was found between aflibercept and ranibizumab in retreatment intervals (SMD=3.97, 95% CI: -0.08 to 8.03, p=0.05) (Figure 12). However, this analysis exhibited high heterogeneity (I²=96%), and with only two studies included, a sensitivity analysis was not feasible. Therefore, these findings should be interpreted with caution.

**Figure 13:**
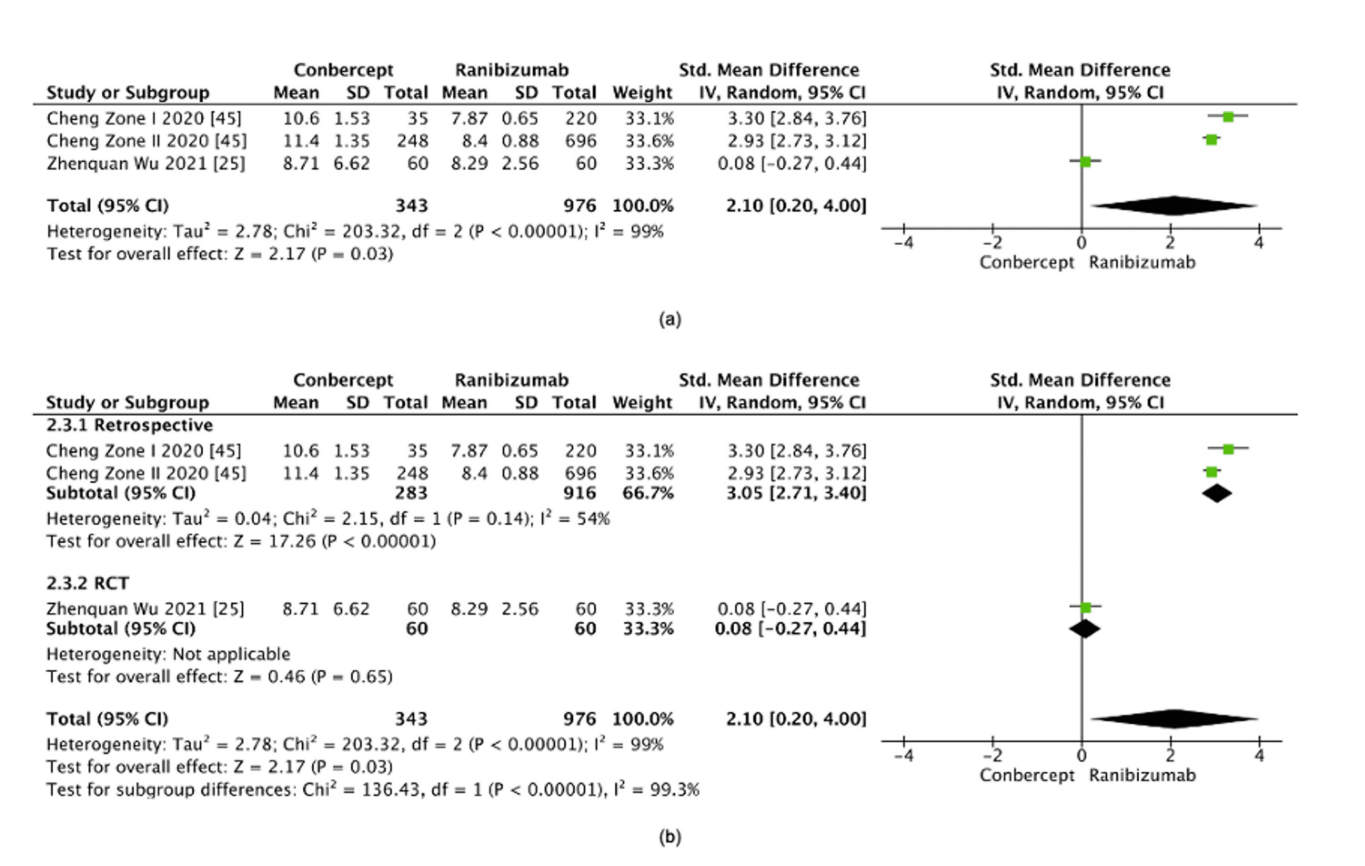
(a) Retreatment interval between conbercept and ranibizumab. (b) Post-sensitivity analysis of the retreatment interval between conbercept and ranibizumab

Conbercept vs. ranibizumab: The comparison between conbercept and ranibizumab demonstrated a statistically significant difference, with conbercept being associated with a longer retreatment interval (SMD=2.10, 95% CI: 0.20-4.00, p=0.03) (Figure 13a). However, this result was accompanied by extreme heterogeneity (I²=99%). Wu et al. [25] was identified as the main source of heterogeneity due to its unique RCT design. A sensitivity analysis which excluded Wu et al. [25] reduced the heterogeneity (I²=54%) and resulted in a stronger effect size favoring conbercept (SMD=3.05, 95% CI: 2.71-3.40, p<0.00001). The final pooled estimate after sensitivity analysis remained statistically significant in favor of conbercept over ranibizumab (SMD=2.10, 95% CI: 0.20-4.00, p=0.03) (Figure 13b).

Successful Retreatment Rates

**Figure 14:**
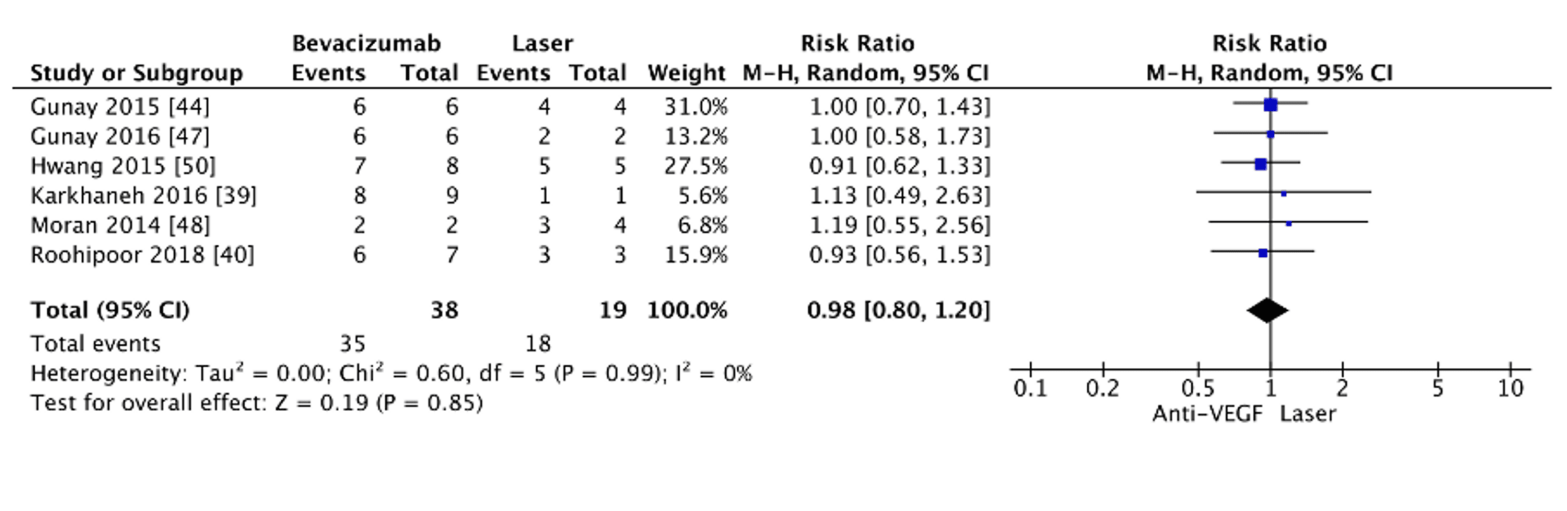
Rate of successful retreatment between bevacizumab and laser

The analysis compared successful retreatment rates where the same treatment modality was used for both the initial intervention and recurrence. Studies that employed cross-over retreatment protocols, such as those by O'Keeffe et al. [34], were excluded to ensure consistency. The pooled results revealed no significant difference in successful retreatment rates between bevacizumab and laser photocoagulation (RR=0.98, 95% CI: 0.80-1.20, p=0.85) (Figure 14). The absence of heterogeneity (I²=0%) suggests consistency across the included studies, enhancing the reliability of these findings. Unfortunately, due to a lack of available data, comparisons with other anti-VEGF agents were not feasible.

Discussion 

Our systematic review aimed to evaluate the recurrence rates of ROP following treatment with anti-VEGF agents compared to laser photocoagulation. Understanding these recurrence patterns is crucial for optimizing treatment strategies and minimizing the risk of ROP recurrence. The initial pooled analysis indicated a significantly higher recurrence rate with anti-VEGF agents compared to laser photocoagulation. However, a detailed analysis of the bevacizumab versus laser subgroup revealed significant heterogeneity (I²=93%), largely driven by the BEAT-ROP trial by Mintz-Hittner et al. [22]. This trial was unique in that it specifically targeted infants with high-risk zone I and zone II posterior stage 3+ ROP. In contrast, other studies included in our analysis, such as Karkhaneh et al. [39], Roohipoor et al. [40], and Linghu et al. [41], encompassed a broader spectrum of ROP severity, including anterior zone II and stage 2 ROP. These differences in study populations likely contributed to the variability in recurrence rates, as less severe cases tend to respond differently to treatment. Furthermore, ethnic and genetic factors may have influenced the recurrence outcomes observed in the BEAT-ROP trial. The study predominantly involved Hispanic infants, while other studies, such as those by Hwang et al. [50], included a more ethnically diverse population. The role of genetic differences in retinal vasculogenesis could potentially impact the effectiveness of anti-VEGF therapy, but this hypothesis requires further investigation, given that most studies did not report detailed ethnic data.

Follow-up duration and the criteria used to define recurrence varied considerably across studies, further contributing to the observed differences in recurrence rates. Studies with extended follow-up periods, such as Linghu et al. [41], were more likely to detect late recurrences, especially in cases where anti-VEGF agents delayed but did not entirely prevent recurrence. In contrast, the BEAT-ROP trial employed a relatively short follow-up period, which may have led to underreporting of late recurrences. Additionally, the definition of recurrence varied between studies, with the BEAT-ROP trial using stricter criteria (reappearance of plus disease or new retinal neovascularization) compared to broader definitions used in other trials. In summary, the discrepancies in recurrence rates between the BEAT-ROP trial and other studies are multifactorial, stemming from variations in patient demographics, inclusion criteria, ethnic composition, follow-up durations, and definitions of recurrence. While the BEAT-ROP trial suggests that bevacizumab may offer a potential benefit over laser photocoagulation, the variability in outcomes across studies underscores the need for more standardized clinical trials to provide clearer guidance on the most effective treatment strategies for ROP. Despite these differences, our pooled analysis from the remaining studies revealed a higher recurrence rate with bevacizumab. Significant heterogeneity was observed in the comparison between ranibizumab and laser photocoagulation (I²=92%). This variability can be primarily attributed to Linghu et al.'s study [41], which reported outcomes that differed markedly from the other studies included in the analysis. Linghu et al. [41] had a considerably larger sample size, with 268 eyes treated with ranibizumab and 111 treated with laser photocoagulation, compared to the smaller sample sizes reported by Zhang et al. [33], Kabataş et al. [52], and Gunay et al. [47]. The disproportionate weight of Linghu et al. [41] in the meta-analysis meant that its results heavily influenced the overall pooled estimates, leading to substantial heterogeneity when its findings diverged from the other studies.

Linghu et al. [41] also differed in their inclusion criteria, encompassing a wider range of ROP severities and treatment zones, including both zone I and zone II. In contrast, the other studies focused more narrowly on zone II. This broader scope likely contributed to the different recurrence rates, as ROP in zone I is typically more severe and may respond differently to treatments like ranibizumab. After excluding Linghu et al. [41] from the analysis, the heterogeneity was entirely resolved (I²=0%), confirming that this study was the primary source of variability. This suggests that differences in sample size, ROP severity, and treatment zones significantly impacted the consistency of findings across the studies. The removal of Linghu et al. [41] provided a more homogeneous dataset, enabling a clearer comparison between ranibizumab and laser photocoagulation, which ultimately reflected a higher recurrence rate with ranibizumab. Additionally, the extent of the recurrence may be influenced by the severity and treatment zone of ROP. Conbercept, a newer anti-VEGF agent, showed a significantly lower recurrence rate compared to laser photocoagulation. This finding is particularly interesting, as conbercept was the only anti-VEGF agent in our analysis to demonstrate a statistically significant advantage over laser photocoagulation. However, it is important to note that this result is derived from a single large study (n=722), and while the sample size is substantial, the lack of multiple studies raises concerns about the generalizability of the findings and limits our ability to draw broad conclusions. The funnel plot generated to assess potential publication bias showed some asymmetry. However, given that the studies were carefully selected based on predefined inclusion criteria, this asymmetry is more likely to reflect differences in study design, populations, and other factors, as identified through sensitivity analyses, rather than publication bias alone.

Recurrence Rate Between Anti-VEGF Agents

Conbercept vs. ranibizumab: Conbercept is a new anti-VEGF agent; it is a recombinant fusion protein composed of VEGFR-1 (second domain) and VEGFR-2 (third and fourth domains) regions fused to the Fc portion of human IgG1 immunoglobulin [35,57,58]. China's Food and Drug Administration has approved conbercept for the management of neovascular age-related macular degeneration and diabetic macular edema. In recent years, conbercept has been used in China for the treatment of ROP, and it has been shown as an effective treatment option [26,59]. In our analysis, conbercept demonstrated a statistically significantly lower recurrence rate than ranibizumab (RR=0.47, 95% CI: 0.39-0.58, p<0.00001). The pooled data from four studies showed no evidence of heterogeneity (I²=0%), indicating consistency across the findings. Conbercept's molecular structure, which binds not only to VEGF-A but also to VEGF-B and placental growth factors (PlGF) 1 and 2, likely accounts for its superior efficacy in preventing recurrence, as it blocks a broader range of factors involved in angiogenesis compared to ranibizumab. The absence of heterogeneity further strengthens the reliability of this result. However, while the current evidence supports the efficacy of conbercept, further research, particularly in diverse populations and with longer follow-up durations, is needed to confirm these findings and assess the long-term outcomes of conbercept treatment.

Aflibercept vs. ranibizumab: Aflibercept showed a lower risk of recurrence compared to ranibizumab, which is likely due to its greater binding affinity for VEGF, as it binds VEGF-A, VEGF-B, and PlGF. Furthermore, the estimated longer half-life of aflibercept (7.13 days), compared to ranibizumab (4.75 days), may account for the lower recurrence rates with aflibercept [60-62]. Moderate heterogeneity (I²=68%) was observed, which should be interpreted cautiously, especially considering the limited number of studies included. Small sample sizes can inflate heterogeneity estimates and reduce the precision of the pooled effect.

Aflibercept vs. bevacizumab: The initial analysis comparing aflibercept and bevacizumab showed no significant difference in recurrence rates, but high heterogeneity (I²=97%) prompted concerns. A sensitivity analysis, excluding Süren et al. [46], reduced heterogeneity considerably (I²=36%) and revealed a significantly higher risk of recurrence with aflibercept. The high heterogeneity in the initial analysis likely reflects variability in follow-up durations and sample sizes across studies. Süren et al. [46] had a relatively small sample size and inconsistently monitored follow-up, which may have contributed to the underreporting of late recurrences. After excluding this study, the remaining data provided a more consistent assessment of recurrence rates. The remaining variability (I²=35%) could be attributed to the disproportionately large bevacizumab arm in Riazi-Esfahani et al.'s study [43], which may have skewed the pooled effect estimates toward the outcomes observed in the bevacizumab group.

Bevacizumab vs. ranibizumab: Despite the initial heterogeneity (I²=78%), the comparison between bevacizumab and ranibizumab ultimately revealed no significant difference in recurrence rates, even after sensitivity analysis. Clinically, this suggests that both agents may have comparable efficacy in preventing ROP recurrence. The absence of a significant difference in recurrence may indicate that other factors, such as dosing regimens, safety profiles, or patient-specific characteristics, are more relevant when choosing between these two treatments. Understanding the differences in the molecular structure between anti-VEGF agents provides insight into their clinical performance. While bevacizumab, a full-length monoclonal antibody, has a longer half-life and more sustained VEGF suppression, ranibizumab, being a smaller fragment, offers quicker clearance but may require more frequent dosing. However, this molecular distinction did not translate into significant differences in recurrence rates in this analysis.

Retreatment Time Interval

Due to the lack of hazard ratio data in the included studies, we used the SMD to assess retreatment intervals. This method allowed for a consistent comparison across different treatment groups. While SMD is not the ideal metric for time-to-event data, it effectively standardized the outcome measures, making it possible to analyze differences in retreatment intervals across these varying study designs.

Bevacizumab vs. laser photocoagulation: Our analysis demonstrated that bevacizumab was associated with a significantly longer retreatment interval than laser photocoagulation, consistent with current literature and bevacizumab's known pharmacokinetic properties [57]. Notably, when we stratified the studies by geographic location, subgrouping them into Ireland, the United States, and Iran, all regions suggested that bevacizumab offered a prolonged retreatment interval. However, geographic factors appeared to influence the extent of this interval. In the subgroup analysis, studies conducted in Ireland, such as those by Moran et al. [48] and O'Keeffe et al. [34], showed a longer retreatment interval, which local treatment protocols, follow-up schedules, or population-specific factors may influence. The studies from the United States, Hwang et al. [50] and Mintz-Hittner et al. [22], similarly supported bevacizumab's longer retreatment time but reported a less marked difference compared to the Irish cohort. The study from Iran, Karkhaneh et al. [39], also found a statistically significant advantage for bevacizumab, but the observed heterogeneity could be partially attributed to differences in patient demographics, healthcare infrastructure, and management strategies between regions. This geographic variation highlights the potential role that local practice patterns, environmental factors, and genetic or ethnic differences might play in determining treatment outcomes. While bevacizumab consistently demonstrated a longer retreatment interval across all subgroups, the degree of this benefit varied between regions. It suggests that while the drug's pharmacodynamics remain constant, other factors, such as healthcare practices and patient-specific variables, likely affect its clinical effectiveness.

Ranibizumab vs. laser photocoagulation: Although ranibizumab was associated with a longer recurrence interval, this finding was derived from a single small trial, Zhang et al. [33], and it was not included in the analysis.

Aflibercept vs. ranibizumab: When compared to ranibizumab, although aflibercept showed a potentially longer retreatment interval, the findings were not statistically significant and were affected by high variability (I²=96%). The current evidence does not support a clear distinction between the two agents regarding retreatment intervals.

Conbercept vs. ranibizumab: Conbercept was associated with a longer retreatment interval compared to ranibizumab. However, the initial analysis exhibited extreme heterogeneity (I²=99%), raising concerns about the robustness of these findings. This variability was primarily driven by differences in study design, particularly between Cheng et al. [45], a retrospective study, and Wu et al. [25], an RCT. Sensitivity analysis, which excluded Wu et al. [22], significantly reduced the heterogeneity (I²=54%) and strengthened the effect size in favor of conbercept. These results suggest that conbercept may be associated with a longer retreatment interval than ranibizumab. Nevertheless, the limited number of studies (n=3) and remaining moderate heterogeneity restrict the generalizability of these findings. Further research is warranted to confirm the potential superiority of conbercept in delaying recurrence.

Successful Retreatment Rates

Our analysis explored differences in retreatment success rates when the same treatment modality was used for both initial and subsequent recurrences. No significant difference was found between bevacizumab and laser photocoagulation, indicating comparable efficacy when reusing either modality for recurrence management. The homogeneity of the data (I²=0%) enhances the reliability of this result, suggesting consistency across the included studies. However, the analysis was constrained by a small sample size, and comparisons with other anti-VEGF agents, such as ranibizumab and conbercept, were not feasible due to insufficient data. This limits the generalizability of these findings to other anti-VEGF therapies. Nonetheless, the results suggest that reusing the same treatment modality, whether bevacizumab or laser photocoagulation, yields similar success rates in cases of recurrence. This information can guide clinical decision-making by reassuring ophthalmologists that retreatment with the same modality, following initial success, will likely produce comparable outcomes for bevacizumab and laser. The exclusion of studies employing cross-over treatment protocols, such as O'Keeffe et al. [34], was necessary to maintain methodological consistency. While comparing similar retreatment modalities and cross-over protocols would offer valuable insights for selecting the optimal retreatment strategy, this analysis was not possible due to limited data. Further research, with larger sample sizes and varied retreatment protocols, is needed to inform clinical practice for managing ROP recurrences.

Limitations

Despite the comprehensive steps employed in this systematic review, there are some limitations which must be acknowledged. These limitations could influence the interpretation and generalizability of the findings but also highlight areas for future research. One of the most significant limitations was the heterogeneity observed in many analyses, especially when comparing recurrence rates between anti-VEGF agents and laser photocoagulation, anti-VEGF agents against each other, and retreatment intervals. The random-effects model was applied in these meta-analyses to accommodate the variability. While some analyses exhibited homogeneity and could have been assessed using a fixed-effects model, this was not pursued, given the overall variability. Although sensitivity analyses identified and resolved some heterogeneity sources, the persistence of variability in some instances suggests that the results should be interpreted with caution. This variability limits the generalizability of our findings. The limited availability of data on some anti-VEGF agents restricted the scope of the analysis. For instance, conbercept's efficacy was primarily based on findings from a single large study, which makes it challenging to draw robust conclusions across diverse populations. Similarly, insufficient data for comparing agents like aflibercept with laser photocoagulation or with other anti-VEGF agents prevented more meaningful comparisons. Differences in follow-up durations among the included studies likely contributed to heterogeneity and affected reported recurrence and retreatment rates. Shorter follow-up periods in some studies may have led to underreporting of late recurrences. This concern was evident in the sensitivity analyses, where excluding studies with unclear or brief follow-up periods significantly reduced heterogeneity.

Another limitation is the inconsistent use of "recurrence" across studies. While some studies defined recurrence based strictly on the reappearance of plus disease, others used broader criteria, including new retinal neovascularization or the need for retreatment. The terms "recurrence" and "reactivation" have also been used interchangeably. Although we predominantly used "recurrence" in our analysis, this definition of variability could introduce heterogeneity and make direct comparisons between studies more challenging. Out of 21 studies in our review, only 12 specified using the ICROP guidelines for diagnosing and classifying ROP, while the rest did not clarify the diagnostic criteria. The ICROP recommends specifying the presence and location of new ROP features by zone and stage; however, not all studies in our analysis provided detailed documentation. Including non-randomized studies increased the study's power and reliability of its findings. While this approach expanded the sample size, it did introduce potential methodological diversity, contributing to the observed heterogeneity. However, most non-randomized studies were judged to be of moderate to high quality based on the NOS. Nevertheless, including such studies could impact the robustness of the conclusions.

Geographic and ethnic differences between study populations were not fully accounted for in this analysis. Several studies were conducted in distinct regions with differing ROP risk factors, healthcare practices, and patient demographics. Additionally, many studies did not report the ethnic backgrounds of their participants. These factors might influence treatment outcomes and recurrence rates, as highlighted by sensitivity analyses. Future research should explore genetic, environmental, and healthcare-related influences on ROP. The analysis employed SMD to compare retreatment intervals due to the lack of hazard ratio data in the included studies. While SMD is a suitable alternative for time-to-event data, the hazard ratio is generally preferred for its clinical relevance. The absence of individual patient-level data limited our ability to calculate hazard ratios, which may have provided more nuanced insights into recurrence and retreatment timing. The review primarily focused on similar retreatment modalities (e.g., bevacizumab retreatment following initial bevacizumab treatment) due to the lack of data on cross-over treatment protocols (e.g., bevacizumab followed by laser photocoagulation). Comparing these protocols could yield valuable insights into alternative management strategies for ROP recurrences, but insufficient data prevented this analysis.

Suggestions for Future Reviews

Future studies should adopt a consistent definition of recurrence in line with the ICROP guidelines. Standardization in diagnostic criteria will reduce variability and enhance comparability across studies. Additionally, incorporating the ICROP's recommended documentation of recurrence, including the presence and location of new ROP features by zone and stage, will help create a uniform understanding of treatment outcomes. Given the limitations of using SMD in the absence of hazard ratio data, future studies should aim to collect individual patient-level data to calculate hazard ratios. This measure is more clinically relevant for assessing time-to-event outcomes like retreatment intervals and could provide deeper insights into the effectiveness of anti-VEGF therapies. The observed geographic and ethnic variability in treatment outcomes suggests the need for further exploration. Future studies should aim to investigate the potential influence of genetic, environmental, and healthcare-related factors on treatment efficacy and clearly document this information.

Subgroup analyses, especially based on treatment zones (e.g., zone I, posterior zone II), would offer valuable insights into the specific efficacy of treatments and for different severities of ROP. Such analyses could help clarify uncertainties, like the higher recurrence rates in zone I treated with laser photocoagulation reported in Mintz-Hittner et al. [22]. Future research should focus on zone-specific outcomes by clearly documenting the zones. The inability to compare cross-over retreatment protocols (e.g., switching from anti-VEGF to laser) highlights a significant gap in current knowledge. Future research should explore different retreatment strategies, such as cross-over protocols, to identify the most effective approach for managing ROP recurrences. The variability in follow-up durations across current studies highlights the need for long-term monitoring to assess the durability of treatment effects. Future research should include extended follow-up periods to capture late recurrences and evaluate the efficacy of treatments over time. This approach is crucial for anti-VEGF agents, which may delay rather than entirely prevent recurrence. Consistent, long-term follow-up data will provide more accurate assessments of treatment outcomes and recurrences. By addressing these areas in future research, we can build a more comprehensive understanding of ROP recurrence.

## Conclusions

Anti-VEGF therapies such as bevacizumab, ranibizumab, aflibercept, and conbercept each show unique clinical characteristics, with differences in recurrence rates and retreatment intervals in ROP management. These treatments serve as an effective alternative to laser photocoagulation by promoting complete retinal vascularization and minimizing the requirement for general anesthesia. However, despite these benefits, anti-VEGF therapy has shown a higher recurrence rate compared to laser photocoagulation. When comparing bevacizumab and ranibizumab with laser photocoagulation, both agents showed higher recurrence rates. In contrast, conbercept demonstrated a statistically significant reduction in recurrence rates over laser photocoagulation, though this finding was primarily driven by a single large study. When pooling data from all anti-VEGF agents, the overall recurrence rate was higher than that observed with laser photocoagulation. When comparing the anti-VEGF agents directly, conbercept demonstrated a significant reduction in the risk of recurrence compared to ranibizumab. Similarly, aflibercept showed a lower recurrence rate compared to ranibizumab. Comparatively, aflibercept had a greater chance of recurrence than bevacizumab, while bevacizumab and ranibizumab showed no significant differences in their recurrence rates. Bevacizumab was consistently linked with a longer interval between treatments compared to laser photocoagulation. This prolonged retreatment interval was observed across different geographic regions, though the extent varied, likely due to geographic, genetic, ethnic, and healthcare-related factors. Conbercept also showed a longer retreatment interval compared to ranibizumab, suggesting its potential to delay the need for retreatment more effectively. However, no significant difference in retreatment intervals was observed between aflibercept and ranibizumab. When looking at retreatment success, both bevacizumab and laser photocoagulation had similar outcomes, suggesting that either approach is equally effective for managing recurrence when used for both initial treatment and follow-up.

## Appendices

**Table 6:** MEDLINE and EMBASE data search history

Structure line	Search terms
1	Retinopathy of Prematurity.mp. or exp retrolental fibroplasia/
2	exp retrolental fibroplasia/ or prematurity of retinopathy.mp.
3	exp retrolental fibroplasia/ or ROP.mp.
4	exp retrolental fibroplasia/ or retrolental, fibroplasia*.mp.
5	exp "Retinopathy of Prematurity"/ or exp retrolental fibroplasia/ or 66etinoopathy* of prematurity.mp.
6	1 or 2 or 3 or 4 or 5
7	Anti VEGF.mp.
8	Anti VEGF injection*.mp.
9	Anti vascular endothelial growth factor.mp.
10	Anti vascular endothelial growth factor injection*.mp.
11	monoclonal antibody.mp. or exp monoclonal antibody/
12	recombinant human* monoclonal immunoglobulin antibody.mp.
13	exp bevacizumab or bevacizumab*.mp.
14	exp bevacizumab/ or Avastin*.mp.
15	exp ranibizumab/ or ranibizumab*.mp.
16	exp ranibizumab or lucentis*.mp.
17	aflibercept.mp. or exp aflibercept/
18	Eylea.mp. or exp aflibercept/
19	exp aflibercept/ or Zaltrap*.mp.
20	pegaptanib.mp. or exp pegaptanib/
21	macugen.mp. or exp pegaptanib/
22	conbercept.mp. or exp conbercept/
23	exp conbercept/ or Luminin.mp.
24	7 or 8 or 9 or 10 or 11 or 12 or 13 or 14 or 15 or 16 or 17 or 18 or 19 or 20 or 21 or 22 or 23
25	recurrence.mp. or exp recurrent disease/
26	reactivation*.mp.
27	exp retreatment* or retreatment*.mp.
28	reactivation interval*.mp.
29	recurrence interval*.mp.
30	25 or 26 or 27 or 28 or 29
31	6 and 24 and 30
